# Correction to: Prevalence of HBV, HCV, and HIV Infections among Patients Undergoing Hemodialysis in Fasa, Iran: A Six-Year Follow-up Study

**DOI:** 10.34172/mejdd.2025.424

**Published:** 2025-04-30

**Authors:** Seyedeh Azra Shamsdin, Mohamad Reza Fatahi, Amir Ansari, Ali Reza Safarpour

**Affiliations:** ^1^Gastroenterohepatology Research Center, Shiraz University of Medical Sciences, Shiraz, Iran; ^2^Department of Internal Medicine, Fasa, University of Medical Sciences, Fasa, Iran

In the article titled *"Prevalence of HBV, HCV, and HIV Infections among Patients Undergoing Hemodialysis in Fasa, Iran: A Six-Year Follow-up Study"* published in *Middle East Journal of Digestive Diseases* (2022;14(3):317-322; doi: 10.34172/mejdd.2022.289), the first name of the third author was incorrect. The correct name is Amir Ansari. This correction has now been updated in both the PDF and HTML versions of the article.

